# Application of CUSUM analysis in assessing learning curves in robot-assisted sacrocolpopexy performed by experienced gynecologist

**DOI:** 10.1186/s12893-024-02691-x

**Published:** 2024-12-04

**Authors:** Kena Park, Ji Young Kwon, Eun-Hee Yoo, Seon Hwa Lee, Jeong Min Song, Seung Yeon Pyeon

**Affiliations:** 1 Department of Obstetrics and Gynecology, Kyung Hee University Hospital at Gangdong, School of Medicine, Kyung Hee University, 892, Dongnam-ro, Gangdong-gu, Seoul, 134-727 Korea; 2 Department of Medicine, Graduate School of Medicine, Kyung Hee University, Seoul, Korea; 3 Medical Big Data Research Center, Research Institute of Clinical Medicine, Kyung Hee University Hospital at Gangdong, Seoul, Korea

**Keywords:** Pelvic organ prolapse, Robot assisted sacrocolpopexy, CUSUM analysis

## Abstract

**Background:**

The aim of this study was to assess the learning curve of robotic-assisted sacrocolpopexy by applying CUSUM analysis based on operation time, complication rate and conversion rate to open laparotomy.

**Methods:**

A retrospective study was conducted with 50 consecutive robotic-assisted sacrocolpopexy surgeries performed from June 2018 and June 2023 by a single experienced gynecologist. Baseline patient demographics, intraoperative parameters and postoperative outcomes were collected. Cumulative sum (CUSUM) of robotic-assisted sacrocolpopexy operation time was analyzed to determine breakpoints between learning phases using piecewise linear regression. This allowed the detection of subtle shifts in surgical parameters and ultimately surgeon proficiency and competency. Continuous variables, such as age, length of hospitalization and op time, were reported as mean (standard deviation). One-way analysis of variance was employed to compare continuous variables. Categorical variables were expressed as percentages and analyzed using the chi-square test.

**Results:**

The regression identified breakpoints at case 8.47 (95% CI 8.0, 9.0) and case 34.41 (95% CI 32.7, 36.1), with an R^2^ value of 0.87, which agrees with that of the second-order polynomial equation. The breakpoints were rounded to the next whole number at case 9 and 35. The *Learning*, *Proficiency*, and *Competency* phases consisted of 9, 26, and 15 cases, respectively in this consecutive series. This suggests that the surgeon achieved proficiency after the first 9 cases and competency after 35 cases. There were no intraoperative nor short-term post-operative complications during the span of this study. Furthermore, there were no conversions to open laparotomy. CUSUM analysis based on complication and conversion rate, therefore, was not available.

**Conclusion:**

According to CUSUM analysis, surgical proficiency of robotic-assisted sacrocolpopexy was attained after the first 9 cases, and stabilization of operation time was achieved after 35 cases. This statistical tool has proven to be useful in objectively assessing learning curves for new surgical techniques, and the transition from laparoscopic sacrocolpopexy to robotic-assisted sacrocolpopexy seems achievable. This, however, may vary with each surgeon’s manual dexterity and experience level. Further investigation with several surgeons and institutions is needed to define a more accurate and generalized learning curve of robotic-assisted sacrocolpopexy.

## Background

Pelvic organ prolapse (POP) affects approximately 40% of women worldwide, and the prevalence is expected to increase with an aging population [1, 2]. Countries in Asia, including but not limited to South Korea, Japan and China, are aging rapidly. This calls for necessary attention to the treatment of POP to improve the health quality of elderly women. Abdominal sacrocolpopexy (ASCP) with mesh interposition has been associated with the highest durability and lowest recurrence of level 1 apical prolapse [3, 4]. However, it is also associated with increased pain, postoperative comorbidities and longer hospitalization. The adoption of laparoscopic sacrocolpopexy (LSC) to alleviate such complications accompanying the laparotomy approach has been limited by a steep learning curve [5, 6]. The superior depth perception and greater dexterity of robotic surgery offers a promising alternative to overcome the obstacles faced during open and laparoscopic approaches to surgically treating POP. Despite these advantages provided by robot assisted surgery, the benefits are uncertain in terms of higher costs and longer operative time [7, 8]. The operative time can vary depending on the surgeon’s competency, patient’s characteristics and coordination within the surgical team. Therefore, studies investigating learning curves based on operative time are important for not only optimizing patient outcomes and deciding cost-effectiveness, but also evaluating feasibility for future unexperienced surgeons. Previous literatures that have studied surgical learning curves for robot assisted sacrocolpopexy (RSCP) have been reported by measuring operative variables, operative outcomes and complications with various statistical methods such as graphical inspection, logistic regression or cumulative sum (CUSUM) analysis [9–14]. Cumulative sum (CUSUM) analysis, a statistical method initially developed for quality control in the manufacturing industry, is able to detect even the subtle shifts in parameters of any given procedure and present a visual representation of the trend as the procedure is repeated over and over again [15]. Applying CUSUM analysis to surgical learning curves can allow real-time monitoring of surgeon proficiency and competency by detecting fine patterns after controlling for random variations [16, 17]. The aim of this study, therefore, was to assess the learning curve of RSCP by applying CUSUM analysis based on operation time, complication rate and conversion rate to laparotomy.

## Methods

This retrospective study included 50 consecutive RSCP surgeries from June 2018 to June 2023 by a single experienced gynecologist. The Institutional Review Board of the Kyung Hee University Hospital at Gangdong approved the protocol for this study (IRB no: 2024-01-025). Data were collected by review of electronic medical records including patient demographics, intraoperative parameters and postoperative outcomes. Basic patient information, such as age, body mass index (BMI), parity, menopausal status, American Society of Anesthesiologists (ASA) score, past medical and surgical history were retrieved retrospectively from medical archives. Intraoperative parameters such as concomitant procedures, total operative time (op time), change of hematocrit (%) and any intra-operative complications were also collected with length of hospitalization and short-term postoperative complications. Total operation time was defined as the time from first incision to that of the final closure.

RSCP was carried out with the aid of da Vinci Xi system (Intuitive Surgical, Inc, Sunnyvale, CA). Three 8-mm robotic trocars and a 12 mm trocar were created. Depending on supply circumstances, two types of mesh: (1) non-absorbable polypropylene mesh (Prolene^®^, Ethicon, Johnson & Johnson, USA) or (2) partially-absorbable (glycolide–co-caprolactone) (75/25) polypropylene-composite mesh (Seratex^®^, Serag-Wiessner GmbH & Co. KG, Germany) were inserted to bridge and fix the anterior and posterior vagina to the sacral promontory. 2 − 0 Polydioxanone (PDS II, Ethicon, Soerville, NJ) suture was used to secure the mesh to the vagina. After incising the peritoneum along the right pelvic sidewall from the sacrum to the cul-de-sac, 1 − 0 Prolene™ polypropylene suture was used to secure the tail of the mesh to the sacral promontory. Peritoneum was then closed with 1 − 0 Coated VICRYL (polyglactin 910) suture to completely cover the mesh.

Assessment of the surgical learning curve was performed using risk-adjusted cumulative summation (CUSUM) methodology in terms of op time and presence of any intra- and post-operative complication. The cumulative sum of the operation times (CUSUM_OT_) was computed for each RSCP surgeries in chronological order by summing the differences between the individual op time (x_i_) and the mean op time (µ) of all cases. The CUSUM at op time n (CUSUM_OTn_) is calculated as follows: CUSUM_OTn_ = $\Sigma _{i = 1}^n\,(xi\, - \mu )$. The CUSUM value for case one represents the difference between its op time and the mean operative time of all cases. Subsequently, the CUSUM for case two is the sum of the difference in op time for case two and the CUSUM of case one. This process is repeated until CUSUM values for all cases are obtained.

Breakpoints in the learning curves were determined retrospectively using piecewise linear regression. A broken-line model was employed to identify case numbers marking transitions between phases of the learning curve, including Learning (Phase 1), Proficiency (Phase 2), and Competency (Phase 3), based on op time. The breakpoints were rounded to the next whole number.

Continuous variables, such as age, length of hospitalization and op time, were reported as mean (standard deviation). One-way analysis of variance (ANOVA) was employed to compare continuous variables. Categorical variables were expressed as percentages and analyzed using the chi-square test. All statistical analyses were performed using the Statistical Package for the Social Sciences (SPSS), version 28.0 for Windows (IBM Corp, Armonk, NY, USA). A p-value of < 0.05 was considered statistically significant. The construction of CUSUM learning curves and piecewise linear regression analysis was conducted using RStudio, version 4.2.2.

## Results

A total of 50 consecutive RSCP surgeries were performed from June 2018 to June 2023 by an experienced surgeon. The baseline characteristics and surgical details of the study population are summarized in Table 1. The study population consisted of 50 females with a mean age of 58 years. Mean BMI was 24.2 kg/m^2^. The number and percentage of participants presented with pelvic organ prolapse quantification (POP-Q) stage of 2, 3 and 4 was 28 (56%), 20 (40%) and 2 (4%), respectively. The mean op time was 222.4 ± 64.3 min, with 45 cases (90%) of concomitant robotic-assisted laparoscopic hysterectomy. The median decrease of hematocrit was 6.5%.

**Table 1:** Baseline patient characteristics and perioperative parameters

Variables	Parameter	Mean, SD	Range	Median
Patient characteristics	Age (years)	57.8, 8.1	38,70	59
	BMI (Kg/m2)	24.2, 2.5	19,31	24.3
	Parity	2.2, 0.6	1,4	2
	Postmenopausal, n(%)	40 (80%)		
	Diabetes, n(%)	8 (16%)		
	ASA, n(%)			
	1	22 (44%)		
	2	26 (52%)		
	3	2 (4%)		
	Previous prolapse surgery, n(%)	9 (18%)		
	Previous anti-incontinence surgery, n(%)	8 (16%)		
	Other abdominal surgery, n(%)	15 (30%)		
	Pelvic organ prolapse quantification stage, n(%)			
	2	28 (56%)		
	3	20 (40%)		
	4	2 (4%)		
Intraoperative parameters	Concomitant procedures, n(%)			
	Total hysterectomy	41 (82%)		
	Subtotal hysterectomy	4 (8%)		
	Bilateral salphingo-ophorectomy	15 (30%)		
	Anti-incontinence surgery	5 (10%)		
	Repair of rectocele	7 (14%)		
	Operative time (minutes)	222.4, 64.3	135,430	204
	Change of hematocrit (%)	-6.4, 2.6	-1.5, -13.4	-6.5
	Presence of uterine pathology, n(%)	30 (60%)		
	Complication, n(%)	0		
Postoperative parameters	Length of hospitalization (days)	5.7, 0.8	5,7	6
	Short-term postoperative complication, n(%)	0		

**Fig. 1:**
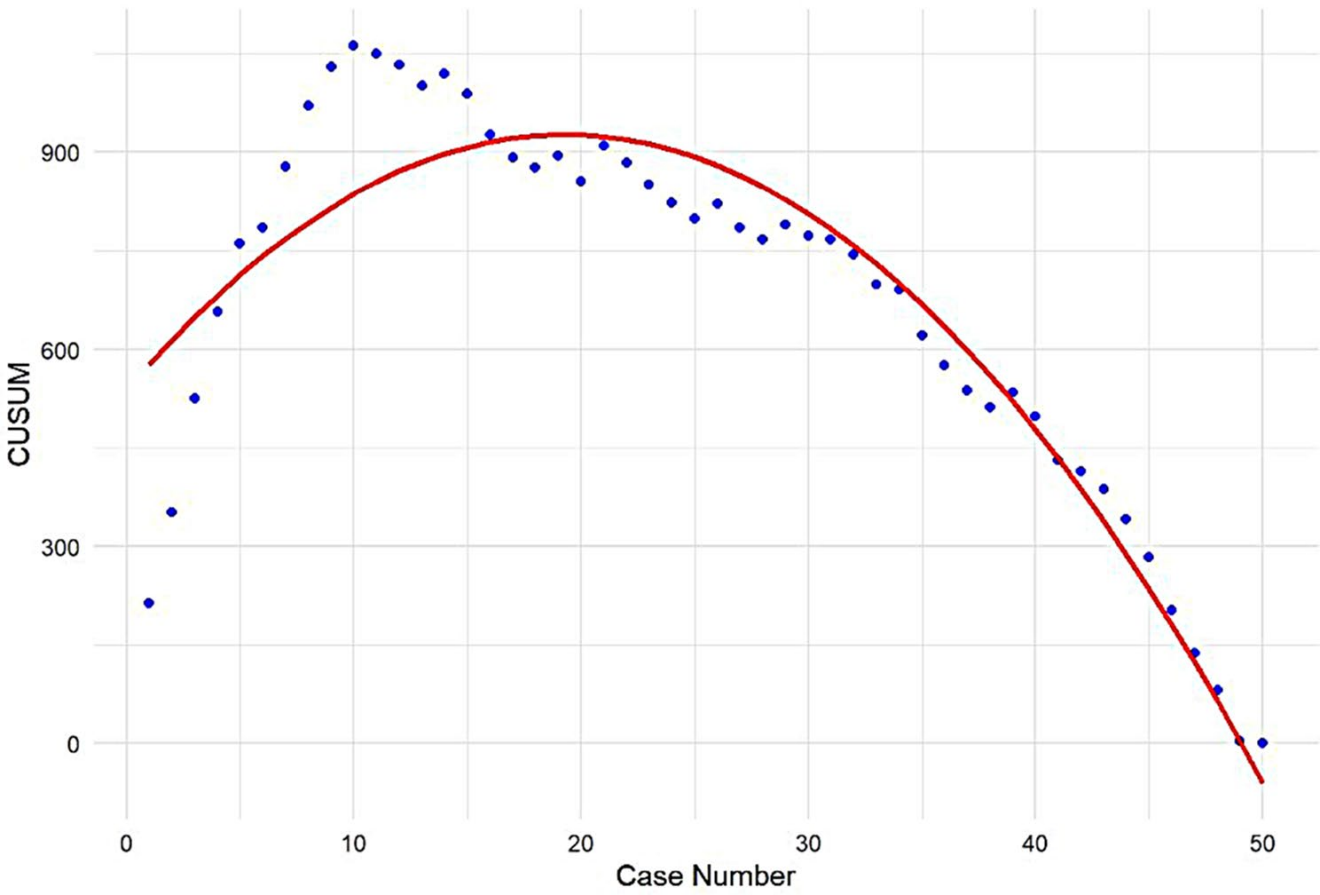
The learning curve (red) of robotic-assisted sacrocolpopexy (RSCP) represented with the best fit curve for the plot, which is a second-order polynomial with the equation CUSUM_OT_ = 668.5 ‒ 1325.5 $\:\times\:$ case number ‒ 1376.9 $\:\times\:$ case number^2^ (R^2^ = 0.87)

**Fig. 2:**
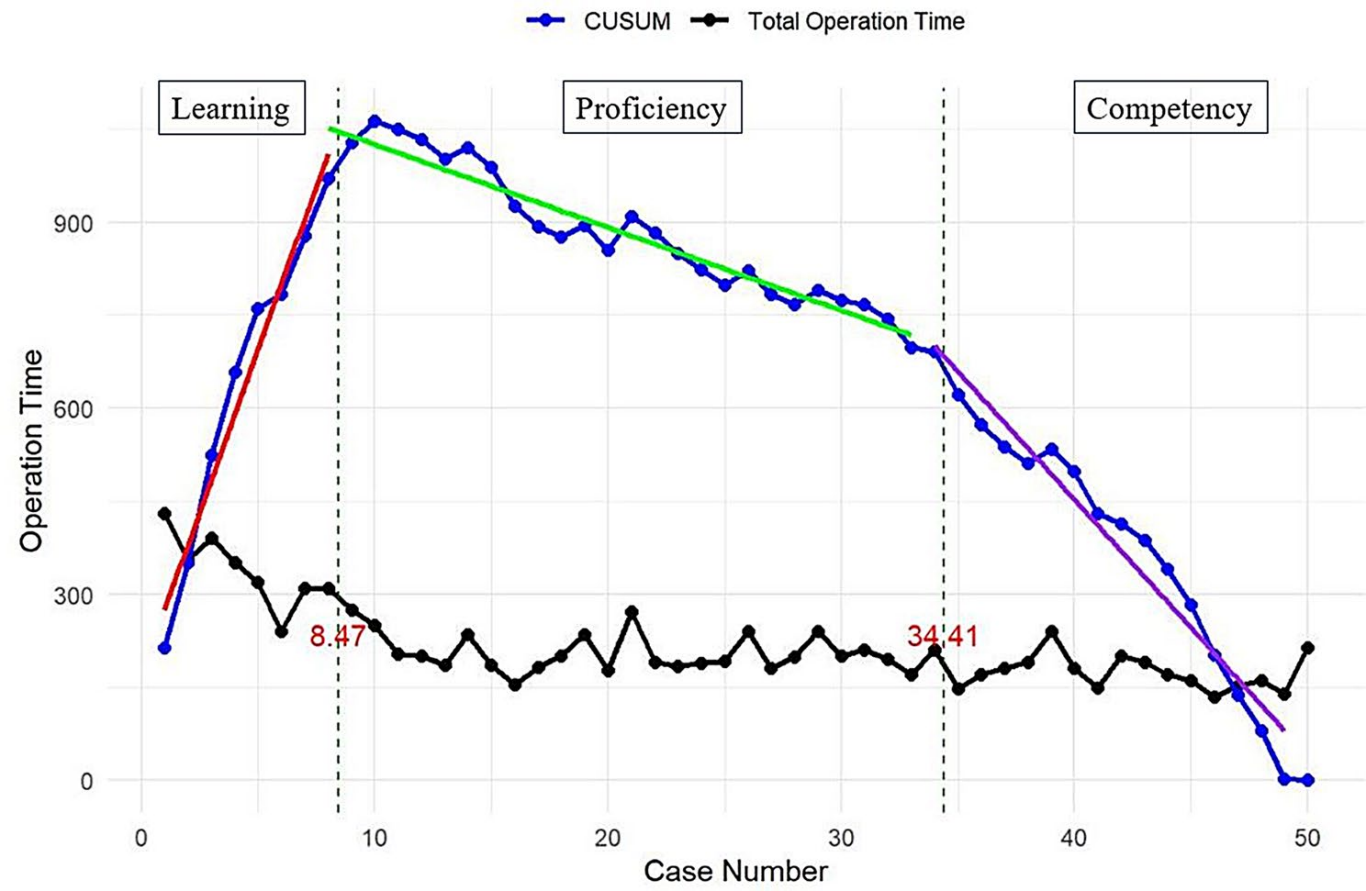
Piecewise linear regression of CUSUM (blue dots) of robotic-assisted sacrocolpopexy (RSCP) operative times (black dots) with breatkpoints at case 8, 95% CI [8.0, 9.0] and case 34, 95% CI [32.7, 36.1], and an R^2^ value of 0.87

The learning curve of RSCP represented with a second order polynomial curve of best fit is shown in Fig. 1. The breakpoints at which the learning phase changes in RSCP op time were determined using piecewise linear regression (Fig. 2). The regression identified breakpoints at case 8.47 (95% CI 8.0, 9.0) and case 34.41 (95% CI 32.7, 36.1), with an R^2^ value of 0.87, which agrees with that of the second-order polynomial equation. The breakpoints were rounded to the next whole number at case 9 and 35. The initial learning curve phase (Phase 1) shows that a surgeon was able to reach the learning phase in every parameter of surgical performance after 9 cases. The subsequent 26 cases led to the achievement of expert competence (Phase 2). The Learning, Proficiency, and Competency phases consisted of 9, 26, and 15 cases, respectively in this consecutive series. This suggests that the surgeon achieved proficiency after the first 9 cases and competency after 35 cases. Comparison of patient characteristics and perioperative parameters among the three phases are summarized in Table 2. The specific mean op time in the Learning, Proficiency, and Competency phases are 338.1 ± 57.4 min, 213.0 ± 34.3 min and 179.9 ± 28.0 min, respectively. A significant decrease in op time was observed between three phases (*p* = 0.000), with a larger discrepancy between the Learning and Proficiency phase. There were no significant differences in baseline patient characteristics among three phases except for the Ba point in POP-Q stage (*p* = 0.005), indicating that op time is not affected by the degree of prolapse in competency phase.

**Table 2:** Comparison of patient characteristics and perioperative parameters according to Learning, proficiency and competency phases

Variables	Learning (1–9, *n* = 9)	Proficiency (10–35, *n* = 26)	Competency (36–50, *n* = 15)	***p*** value
Op time (minutes)^a^	338.1, 57.4	213.0, 34.3	179.9, 28.0	0.000
Age (years)^a^	53.2, 7.8	57.9, 7.6	59.6, 8.7	0.193
Parity^a^	2.5, 0.5	2.1, 0.6	2.1, 0.6	0.258
BMI(kg/m^2^)^a^	23.8, 0.8	24.7, 3.0	23.5, 2.1	0.273
Menopause	5/8(62.5%)	21/26(80.8%)	14/16(87.5%)	0.349
Diabetes	1/8(12.5%)	6/26(23.1%)	1/16(6.3%)	0.337
Hypertension	1/8(12.5%)	13/26(50%)	5/16(31.3%)	0.128
Ba^a^	0.5, 1.2	1.0, 1.1	2.4, 1.6	0.005
Bp^a^	-1.9, 0.4	-0.6, 1.8	-0.9. 2.6	0.259
C/D^a^	0.4, 1.3	1.3, 1.7	1.7, 2.1	0.248
TVL^a^	7.3, 1.3	7.3, 0.8	7.9, 0.8	0.218
POP-Q stage^b^ (2-3-4)	6-3-0	13-11-2	9-6-0	0.546
ASA^b^ (1-2-3)	6-3-0	11-13-1	5-10-1	0.270
Prior abdomen-pelvic surgery^b^ (0-1-2)	6-1-1	18-7-1	11-5-0	0.582
Prior prolapse surgery	2/8(25%)	4/26(15.4%)	3/16(18.8%)	0.822
Prior anti-incontinence surgery	3/8(37.5%)	2/26(7.7%)	3/16(18.8%)	0.124

^a^expressed as mean ± standard deviation, ^b^expressed as number in section

There were no intraoperative and short-term post-operative complications during the span of this study. Furthermore, there were no conversions because of robotic surgery failure. CUSUM analysis based on complication and conversion rate, therefore, was not available.

## Discussion

For the interpretation of our CUSUM results, it is important to realize the downward slope indicates a shorter op time than expected while an upward slope indicates a longer op time than expected. A prolonged upward trend may call for further investigation and identification of possible reasons behind the sudden increase in op time. Our data shows a plateau in op time after first 8 cases. This is followed by a steep reduction in CUSUM of RSCP after 35 cases. Proficiency was thus achieved by case 9, and competency was achieved by case 35. In the competency phase, op time is not affected by the degree of prolapse. Aside from the decrease in op time, several other variables can be used for monitoring and auditing surgical performance such as estimated blood loss, pain medication and hospital stay. However, no significant statistical differences were noted among three phases (data was not shown). Moreover, no peri- and short-term post-operative complications were reported during this study.

There are very few published papers about the learning curve of RSCP, and analysis methods even differed from one another. By graphical inspection of op time, Akl et al. reported 25.4% decrease of op time after the first 10 cases, with the last 30 cases having a mean op time of 167.3 min [9]. Geller et al. reported decline in op time by > 1 h after first 10 cases with a median op time of 254 min of the remaining 137 cases. A significant decline after 20 cases for critical steps of the procedure was observed, represented by an inflection point of considerable reduction in performance time at 60 cases by the split group method (dividing the data into consecutive groups and comparing group means) and the cubic function of fitting smoothing curve analysis [10]. Myers et al. applied CUSUM to monitor, not a learning curve but, maintenance of proficiency at the target value of a 10% complication rate [11]. Linder et al. reported that median op time plateaued after first 60 cases from 5.3 to 3.6 h. Proficiency, as determined by a risk-adjusted CUSUM analysis for complication rate, was achieved after approximately 84 cases. The rate of intraoperative or grade 2 + postoperative complication was reported to be 26.8% [12]. Sharma et al. reported that proficiency was noted at 25 cases and efficiency at 36 cases and no significant improvement in op time after 60 cases with mean op time of 247 min after 36 cases by the B-spline regression and sequential grouping model for op time [13]. Van Zanten et al. reported that op time dropped after 20–24 cases and stabilized between 24 and 29 cases, and mean op time was 173 min. The proficiency based on CUSUM analysis of the rate of intraoperative complications was obtained after 78 cases. The rate of intraoperative complication was reported to be 1.9%.

Existing literature investigating the learning curve of RSCP is limited, and outcomes were analyzed with different definitions and confounding factors such as discrepancy in prior surgeon experience was not eliminated. This presents a challenge to draw on concrete findings and conclusions based on the literature so far. The importance of understanding the learning curve and establishing a surgical training program to enable safe and effective surgery cannot be overemphasized. And the first step in doing so is reaching a consensus on a standardized reporting system to standardize outcome.

CUSUM analysis has potential to be adopted as a standardized self-monitoring tool in assessing learning curves of surgical procedures due to its ability to efficiently detect and visualize subtle trends in parameters. However, it is also important to be aware of its limitations. The most blatant limitation lies within its strength: CUSUM is primarily effective for merely detecting trends and shifts in performance without the ability to provide insights into the underlying reason for changes such as patient selection, trainee involvement or concomitant procedures [12, 14]. This leaves room for misinterpretation, leading to incorrect conclusions about a surgeon’s proficiency. Risk-adjusted CUSUM analysis may serve to compensate for CUSUM’s inability to account for variability in case complexity.

Previously reported numbers for proficiency are considerably higher than our results. The discrepancy in surgeon experience and individual skill set is a possible explanation. This study monitored 50 RSCP procedures performed by a single gynecologist with plentiful cases of surgical experience. This explanation of proficient surgical experience also offers an explanation to the absence of intraoperative complications. Also, it was possible to get a triphasic CUSUM curve with inflections at case 9 and 35 through overall operative time. The completion of all procedures by a single surgeon bestows homogeneity and is a strength of our study. However, a single surgeon may also be viewed as a limitation due to the lack of generalizability.

Additional limitations include the retrospective study design and thus the lack of specific segmentation of each step such as docking, console, concomitant operative and suture times. Concomitant procedures such as total and/or subtotal hysterectomy, salpingo-oophorectomy, anti-incontinence surgery and rectocele repair were included in total operative time. Concomitant operative times, compared to RSCP opearative times, occupy a minor span of the total operation time. However, this challenges the homogeneity of the data and must be taken into consideration. Operative time is also reflective of harmony of several factors of operative platform, surgical teams including assistant and anesthesiology. Rather, overall op time might be better indicator of surgical proficiency.

## Conclusion

In conclusion, according to CUSUM analysis, surgical proficiency of RSCP was attained after first 9 cases, and stabilization of operation time was achieved after 35 cases. This statistical tool has proven to be useful in objectively assessing learning curves for new surgical techniques, and the transition from laparoscopic sacrocolpopexy to RSCP seems achievable. This, however, may vary with each surgeon’s manual dexterity and experience level. Further investigation with several surgeons and several institutions are needed to define a more accurate and generalized learning curve of RSCP.

## Data Availability

The datasets on which the conclusions of the manuscript rely are all presented in the manuscript.

## Abbreviations

POP: Pelvic organ prolapse
ASCP: Abdominal sacrocolpopexy
LSC: Laparoscopic sacrocolpopexy
RSCP: Robot assisted sacrocolpopexy
CUSUM: Cumulative sum analysis
BMI: Body mass index
ASA: American Society of Anesthesiologists
Op time: Operative time
ANOVA: One-way analysis of variance
SPSS: Statistical Package for the Social Sciences
POP-Q: Pelvic organ prolapse quantification
